# Single Mothers’ Experiences with Pregnancy and Child Rearing in Korea: Discrepancy between Social Services/Policies and Single Mothers’ Needs

**DOI:** 10.3390/ijerph15050955

**Published:** 2018-05-10

**Authors:** Jung-Eun Kim, Jin Yong Lee, Sang Hyung Lee

**Affiliations:** 1 Division of Social Welfare Policy Coordination, Ministry of Health and Welfare, Seoul 03741, Korea; lydiasilver@gmail.com; 2 Public Health Medical Service, Boramae Medical Center, Seoul National University College of Medicine, Seoul 07061, Korea; 3 Institute of Health Policy and Management, Medical Research Center, Seoul National University, Seoul 03080, Korea; 4 Department of Neurosurgery, Boramae Medical Center, Seoul National University College of Medicine, Seoul 07061, Korea

**Keywords:** single mother, healthcare, childcare, employment, housing, self-reliance

## Abstract

This study aims to explore single mothers’ experiences with social services/policies for their independent living and to identify gaps between these experiences and the needs of single mothers. A focus group discussion was performed to collect data. Seven single mothers discussed their experiences in significant periods of their lives: pregnancy, childbirth, and parenting. Findings from the qualitative thematic analysis show discrepancies between the direction of social services/policies and single mothers’ needs, in terms of difficulties in healthcare, childcare, housing, employment, and income security. To the single mothers in this study, the social safety net is not inclusive, compared to that which is available to two-parent families or adoptive families. It is necessary to intervene in current blind spots of services/policies for single mothers, and to provide a social safety net to strengthen single mothers’ self-reliance and their children’s social security in the long term.

## 1. Introduction

Single-parent families are increasing around the world. Fifteen percent of children worldwide live in single-parent families and most of these households are headed by women [1]. Women-headed households are more likely to be in danger of poverty, which could result in worsening inequalities in health [2]. There were approximately 35,000 single mothers ((1) in Appendix A) in the Republic of Korea (South Korea) in 2010 and this number has been increasing since 2000 [3,4]. This increase is a result of single mothers being willing to raise their children rather than give them up for adoption [5,6]. This reflects societal change, because previously, single mothers in South Korea were viewed as transgressive as they went against social norms, social order, and hegemonic family structure. Under these social norms, most babies born to single mothers were sent abroad for adoption [7].

However, Korean society has started to accept the responsibilities and rights of single mothers as decision makers about pregnancy and childbirth [6]. For example, the Single-Parent Family Support Act (SPFSA) was enacted in 2007 to support single parents in achieving social and economic independence through the provision of health security. The Act includes the support of welfare vouchers for single parents to supplement their medical and childcare expenses and provides funding to single-parent family shelters. However, it only guarantees a minimum living standard ((2) in Appendix A) for single-parent families that are either below the poverty line or headed by someone younger than 24 years old. Indeed, the policies have missed the increasing number of single mothers in their late 20s or 30s who show a relatively higher willingness to raise their children than single teenage mothers ((3) in Appendix A). The single mothers in these age groups often have jobs or have prepared to get a job, prior to childbirth. However, the unintended pregnancy and failed relationship with their child’s father often makes them more vulnerable to challenges such as job loss, forgone higher education opportunities, and broken relationships with their original family and friends. In addition, the social benefits focus more on the period of pregnancy when single mothers stay in shelters, rather than their life after childbirth, outside the shelters.

In Korean society, social policies and services for single mothers remain underdeveloped to respond to changing family structures and social norms related to single mothers’ needs. In this context, this study aims to understand single mothers’ experiences of policy changes and the gaps that need to be addressed in the future to help mothers achieve better, independent lives.

## 2. Methods

### 2.1. Study Setting

Focus group discussion (FGD) was used to collect data. Initially, we contacted six recognized single mother shelters in Seoul and visited them after obtaining the necessary permissions. We met the executive directors of each shelter to share the purpose of our study and to understand the current services that were provided to single mothers by the shelters. During the six months, we tried to build a relationship of trust with the organizations and then incorporated their comments and advice in our study. Finally, only one shelter connected us to a reunion social support group of single mothers. Among these members, we selected voluntary participants and conducted one FGD with seven single mothers. We considered in-depth personal interviews with each single mother. However, we realized that individual interviews were not a comfortable setting for single mothers, as we did not recruit volunteer interviewees for a long period. Members of one self-help group for single mothers suggested that they could participate in the study if we offered a group interview instead of individual interviews. Thus, we decided to conduct focus group discussions. We believed that adopting FGD as the main study method was a good choice. During the FGD, participants were able to talk to each other in a comfortable environment; they laughed and cried and showed each other empathy (Table 1).

### 2.2. Data Collection

We developed a semi-structured questionnaire with a focus on three significant periods of a single mother’s experience: pregnancy, childbirth, and parenting. The questions were designed to cover the history of the participants’ pregnancy and first childbirth, and then focused on parenting issues, including experience with infant care, early childhood, and future parenting concerns. The moderator used the questionnaire to lead the discussion during the FGD. The FGD was conducted for five hours in an observation room equipped with audio recording equipment at Gallup Korea. We had a break every hour or when participants requested them. We observed the participants from a room with a one-way mirror, with their permission.

### 2.3. Participants

Seven single mothers participated in the FGD. We chose participants who were raising children and living in the community during the past ten years, which is the significant period for policy change for single-parent families in Korea. We used these criteria to select participants because they had made the choice to keep their children over other options such as abortion and adoption. Table 2 shows the general characteristics of FGD participants.

### 2.4. Data Analysis

The audio recordings from the FGD were transcribed verbatim into an electronic transcript in Korean. After analysis, the Korean transcript was translated into English language for this paper. This study was designed to present findings as patterns of interrelated concepts within the dataset [8,9]. Specifically, we employed a qualitative thematic analysis of a focus group discussion, which allowed us to identify data patterns with specific meanings and potential implications relating to the research topic [9,10]. We began with line-by-line initial open coding of the data. The initial codes were grouped under axial coding as common categories based on code similarity and relatedness. Then, we searched potential themes from the categories and codes, and checked if the themes were conceptually defined by the categories and generalized across the dataset. We selected clear extract examples by key themes, related to our original research interest and FGD questions. To increase reliability and validity of the data analysis, the two coders conducted a reiterative comparison review by merging key themes from the coded content. In addition, the research team also reviewed the coded themes and held discussions with the coders to reach consensus regarding the key study findings. We did not use a specific software for the analysis; instead, we used MS Word functions such as text highlighting and insertion of memos. We incorporated investigator triangulation in the data analysis by employing multiple researchers from different fields such as medicine, social work, and nursing. This researcher triangulation increased data validity by providing multiple perspectives of one focus group discussion.

### 2.5. Ethical Considerations

After confirming the participants of the FGD, the participants were sufficiently informed about the study’s purpose; they understood that no harm would be caused. Their privacy and personal information were protected with written informed consent. An incentive (around $90) was given to each participant after the FGD. The study was conducted in accordance with the Declaration of Helsinki, and the Institutional Review Board of Seoul National University Boramae Medical Center (IRB No. 26-2014-132) approved this study.

### 2.6. Study Limitations

This study has limitations regarding its method and design. The sample included single mothers in their 30s raising a young child alone who were self-reliant and came from the common context of conservative familism in East Asian countries. However, the sample could have differed from single mothers who gave a birth as young teenagers, stopped higher education, chose adoption after childbirth, or became married. Because we did not use comparative data analysis and did not employ additional focus groups for saturation, the generalizability issue remains. Further studies with additional focus group interviews and quantitative studies can validate the findings.

The FGD as the main study method was appropriate to identify common or different experiences with social services for the single mothers these policies targeted. If the single mothers experienced different levels of quality in social services, we could explore blind spots of the policy/service for single mothers. However, during the group discussion, participants might have been concerned about unwanted attention or confidentiality regarding other participants or researchers. As a result, the group discussion could miss personal details regarding childbirth and parenting, such as mental health and relationship problems with family or the child’s father.

## 3. Results

**Figure 1:**
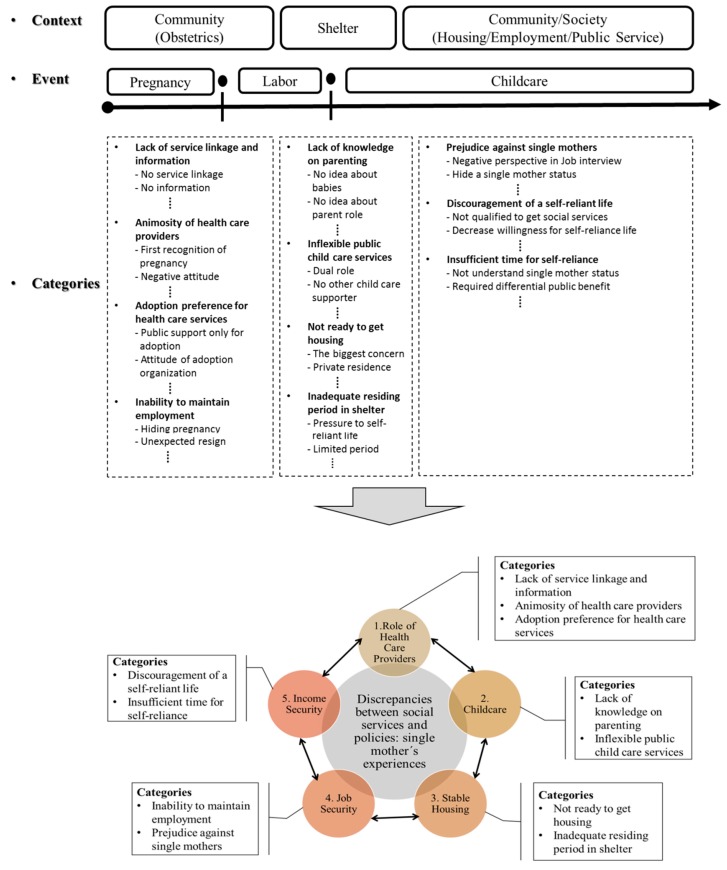
Diagram for study framework according to three significant periods and five main themes.

The researchers identified 52 codes from the FGD scripts and then merged the raw codes into 11 subcategories. The subcategories were further merged into five main themes. The final key themes that emerged were role of health care providers, childcare, stable housing, job security, and income security (Figure 1). Further detailed explanation by theme is presented below, with representative quotes of participants. We attached quotes when at least three participants mentioned an issue and confirmed unanimous perspectives in the group discussion.

### 3.1. Theme 1: Role of Health Care Providers

The first theme was regarding role of health care providers, especially during pregnancy. The participants expressed their confusion and difficulties in choosing abortion or childbirth when they were told they were pregnant. It was very difficult to obtain any information about pregnancy and child rearing at hospitals or obstetric clinics. The single mothers also reported that they experienced “cool vibes” from healthcare providers.

Participant 2: *When I first visited the obstetric clinic and told the obstetrician that I was not married, the hospital suggested abortion. They did not provide any information or other options for me besides abortion.*

The single mothers also reported that they found the health care providers to be unhelpful and judgmental. In addition, when they contacted social service agencies for support during childbirth, the services and financial aids for childbirth were limited, except for mothers who chose adoption.

Participant 3: *I think the perception about single mothers is very problematic. When I visited hospitals and public health centers to submit proof of employment, one staff member said he needed the father’s document as well. Therefore, I told them that I was a single mother. My daughter was standing beside me. The staff member said, “does a single-parent family not have a father?” At that moment, I felt very ashamed and upset.*

Likewise, attitudes and perspectives of health care providers were important to the single mothers to determine the quality of health care services. Since the hospital and public health centers were the first social services accessed by the single mothers, experiences with the health care providers were a key determinant for the single mothers to continue to seek help from society.

### 3.2. Theme 2: Childcare

The participants were undergoing difficulties in raising their children. They had limited time to consider how to raise their children by themselves prior to childbirth because they had unexpected pregnancies. They reported that they did not have sufficient knowledge about parenting. Specifically, they had insufficient resources to obtain information on parenting because most of them did not have the support of their families and were isolated from their friends and colleagues. Therefore, they had less social support for parenting as compared to two-parent families. The single mothers needed to obtain more information about public childcare services because of their lack of knowledge and resources on parenting.

Participant 5: *I searched for the term “single mom” on the internet, as it was very strange to me at that time. I searched for the term “parenting alone” as well. I found some information there…*

Participant 7: *I did not know that the baby does not sleep in the night. I did not know that they keep waking up. Before childbirth, I just knew that the baby always lies down, sleeps, and needs milk.*

Participant 6: *When I entered the shelter, I was young and had just learned how to bottle-feed rather than breastfeed. I had no knowledge about how to raise a baby. I found some information via the memos on the mother’s check-up planner. My breasts were very swollen, so I learned first how to breastfeed in the shelter.*

The participants experienced inflexible public childcare services, because there were no childcare centers to take care of children at night or on weekends near their workplaces or homes. If a mother wanted to leave her child in a hurry because of shift work or a job interview, there was no way to hire private childcare providers.

Participant 3: *If I have to work at night unexpectedly, I usually take a taxi to pick up my daughter and then go back to my office.*

The parenting experiences of these single mothers might be similar to those of two-parent families in Korean society, for whom a lack of public childcare services and higher responsibility of mothers for childcare are reasons for the lower birth rate. Nevertheless, single mothers cannot confidently request or receive public childcare services or family support for childcare, because they are unwed mothers.

### 3.3. Theme 3: Stable Housing

When the single mothers decided to raise children on their own, their biggest concern was housing. Even though the Korean government gives single mothers public housing priority, maintaining eligibility is very difficult. To maintain eligibility for public housing, mothers must work and earn money as soon as possible. However, ironically, if their income is higher than the minimum threshold of the national poverty line by even $1, they are not eligible to receive public financial support. Single mothers slightly above the poverty line were more likely to shift houses, depending on their economic condition, without considering factors such as safety of the community and distance from day care centers and their place of work.

Participant 7: *The real world was… I thought that the shelter would solve my issues but the shelter said that I have to leave within 3 months. Therefore, I moved to another shelter and stayed there for 2 years. There was no house I could go to after that. I stopped by several single mother shelters in different communities and finally settled in B community. However, it is still very hard, especially for my baby because we frequently changed day care centers…*

The single mothers had to find a new residence for themselves and their children within 100 days, because that was the maximum duration that they were permitted to stay at the shelters after childbirth. However, it was difficult to find new housing while managing their financial condition right after leaving the shelters.

### 3.4. Theme 4: Job Security

Employment is a significant concern for single mothers; the participants faced many challenges while seeking employment or keeping their jobs because they were single mothers. They were unable to tell their coworkers or company about their pregnancy, so they could not access maternity leave and had to quit their jobs when they decided to have the child.

Participant 1: *It was very hard to seek employment during pregnancy. I was wrong to think I could work even during pregnancy. I could not receive a proper salary during pregnancy. My financial plan was not successful, and I could not get severance pay either…. One of my friends recommended that I enter the shelter…*

After childbirth, the single mothers tried to search for jobs, but they were discriminated against because of the stigma attached to having a baby outside of legal marriage. They thought that hiding their identity as a single mother was better for maintaining a stable job. This finding shows that Korean society still has negative perceptions toward women who have children outside of marriage.

### 3.5. Theme 5: Income Security

The current social policy and services for single parents aim to help them secure sufficient income for them to sustain an independent life. However, participants perceived that the policy made them more dependent on public assistance. Some single mothers were not willing to seek jobs, despite being capable of working, just so that they could meet the eligibility criteria for receiving public assistance. If the working single mothers’ income level was not below the poverty line, they would be excluded from the social safety net.

Participant 2: *I think the policy should be period-based, not finance-based. As a single mother, it might take me 10 years to develop a self-reliant life. I do not think I can earn so much money. If the policy can provide me with public support for a longer, finite period, I can pay back to the nation with the saved money I collected during the period where the policy covered me. I mean, I hope that the policy can secure sufficient time for me to achieve a self-reliant life, rather than extend support based on my current financial status. During that time, I can get a job and earn more money and eventually not require public assistance…*

The status of being a single parent was not a sufficient condition to receive public assistance. Even if the single mothers had jobs, their job condition was likely to be unstable with an income near the poverty line.

## 4. Discussion

The Korean government has recognized the necessity of policy intervention for single mothers and has tried to support this vulnerable group as a part of family support policy or poverty policy. The primary goal of the government effort was to support them in achieving social and economic independence and health security. However, we confirmed that there are discrepancies between social services/policies and single mothers’ needs. To the single mothers in this study, the social safety net they experienced was not inclusive, compared to that received by the two-parent family or adoptive family. Thus, we found that Korean society for single mothers is still very conservative.

### 4.1. Key Role of Health Care Providers as the First Contact Point of Single Mothers

Health care providers or public officers for social services are the first public point of contact of single mothers. A woman with an unexpected pregnancy starts to be called a mother by her health care provider based on a medical exam and her physical condition. The government provides low-income or younger single mothers with coverage for medical expenses in the context of their health security, because medical check-ups for single mothers until childbirth are essential. From our findings, we understand that the role of health care providers is very important for providing information and consultation about how to be a good mother. Depending on how the first meeting with the health care provider goes, the woman may become a good mother, or give up on being a mother. Nevertheless, current social policy has ignored the importance of educating healthcare providers to respect mothers-to-be, regardless of their marital status and socio-economic position. Health care and social service systems should create an environment where single mothers feel safe and supported and work towards enabling them to fulfill their responsibilities as mothers.

### 4.2. Barriers to Accessing Childcare Services, Doubled by the Burden of Childcare Expenses

After childbirth, the participants of the study took on dual roles, as breadwinners and caregivers, and had difficulty maintaining an independent life. Single mothers with relatively unstable jobs face difficulties with childcare as well. They are not able to hire private babysitters and to receive assistance from public childcare systems when they are looking for jobs or are in job training. To meet diverse childcare needs, the Korean government recently increased the number of 24-hour day care centers and ensured that some centers remain open on holidays and weekends. However, this is only 1% of the total childcare centers in Korea [11]. The national childcare system provides 600 hours ($10 per hour) of childcare services per year, which can be used at night, on weekends, or holidays with an additional fee ($5 per hour). The level of government support for the total service fee varies by the income level of a household, and the parent share of the fee is still an economic burden. High competition for spots in public childcare centers, long wait times, and the burden of service fees childcare might exclude single mothers’ children from the public safety net. The magnitude of the stress the single mothers experienced regarding childcare was also doubled by the economic burden for single parents.

### 4.3. A Lack of Affordable Housing Considering Needs of Single-Parent Families

Housing is a significant issue during pregnancy and after childbirth [12]. It is necessary for single mothers to manage their own accommodation independently, given that they chose to keep the baby. However, this basic condition necessary to secure a healthy environment is difficult for them to achieve because of their financial situation. Current policy for single-parent families prioritizes single mothers while allocating public housing, but mothers still had difficulty meeting the minimum financial criterion for eligibility and could not find a stable source of income to pay the rent. The national credit loan with a lower interest rate for tenancy deposit is targeted at newly married couples or families with multiple children. In addition, the poor (less than 40% of the median income) are eligible to receive housing expenses from the government. However, housing support for single-parent families is lacking. Non-profit shelters for single-parent families are the only residence safety net for these mothers (maximum 1-year stay). The participants of the study suggested that the policy should provide them with sufficient time to live independently as well as to relax the financial criterion to be eligible for public housing. To meet these needs, the Korean government initiated the development of a housing support project for single-parent families in 2015. The housing project was only for single-parent families and planned to provide group homes for at least 2 years, with a rent less than $100 per month. The Ministry of Gender Equality and Family supports the lease deposit after confirming a parent’s willingness and ability to achieve self-sustainable living. Despite good trials of this housing policy, so far only 50 houses have been provided nationwide.

### 4.4. Lower Acceptability of Work Environment for Single Mothers 

Another difficulty as a single mother is maintaining a stable job and suffering prejudice since the Korean society stigmatizes women who have children outside of marriage. Such a stigmatized social status is a significant stressor for mothers and affects their economic activities and parenting. Korea launched a system to certify family-friendly companies in 2008 to guarantee workers’ comfortable childbirth and parenting. A parental leave (1 year) or maternity leave (30–60 days) is guaranteed by law in Korea. However, these policies are oriented for two-parent families and full-time positions, and single mothers cannot use parental leave because of their unmarried status. If they want to use maternity leave, they have to quit their job. If a young single parent wants to continue her studies or participate in job training after childbirth, the Korean government provides her with education expenses in addition to childcare expenses. However, single mothers over 25 years old cannot receive any social support to re-enter education or employment. Thus, we confirmed that single mothers are excluded from Korea society even when Korean society is trying to increase the birth rate by promoting employment security.

### 4.5. Weak Financial Incentives for Income Security

The final theme that emerged from our findings was income security. We assert that support for the four themes described above, role of health care providers, childcare, stable housing, and job security, will strengthen income security by promoting single mothers to become self-reliant. In a review of child raising expenses for single-parent families from the government, when the monthly income of single mothers was higher than 52% of the median income in a relevant year, they were excluded from public assistance, the only cash benefit from the government. These welfare benefits for childcare for single parents cannot be duplicated with the living expenses for the poverty group (less 30% of the median income). Thus, raising a child without a job rather than earning money can secure higher income from the government as well as sufficient time to take care of children. A single mother with “poor” status is eligible to receive nearly $800 per month, but a working single mother can receive only $130 when her income level is not higher than 52% ($1400) of the median income. In recent statistics, over 50% of single mothers were part-time or shift workers, and their average household income was less than $1000 per month [4]. We assume that such a small cash benefit ($130) does not work as a positive attraction for single mothers’ self-reliant living (Table 3).

Moreover, existing policy for single parents focuses on secondary or tertiary family assistance, such as adoption and institutional childcare. For example, the child benefits from the government for adoptive families is $150 until the adopted child is 16 years old. Children living in shelters are supported by the government with approximately $2000 per month, including living expenses, educational assistance, and additional administrative expenses for the institution. Current policy provides less financial support to working single mothers, even though they are more likely to be unemployed and experience financial difficulties. A policy redesign is required to support working poor single mothers in becoming self-sufficient, which can prevent social isolation and promote the well-being of the child [13,14,15].

We found an unintended consequence of the single-parent family policy was that single mothers were less likely to find a job, as maintaining poverty status enabled them to meet criteria to receive public and private assistance. The existing single-parent family policy discourages single mothers from seeking employment, because of the small difference between the salary obtained from unstable employment and the financial assistance provided by support services. This finding is similar to the results of previous studies on the ineffectiveness of single-parent family policy in the United States, which showed that the income received by single mothers in the labor market could not cover the cost of standard of living and child rearing [16,17]. To supplement such limitations of the policy design, the Korean government adapted the Earned Income Tax Credit in 2008 to support the working poor. In 2018, the Korea EITC for single-parent households was a maximum $2000 per year when the total annual household income was less than $9000. Thus, the range of tax benefit is still narrow to promote higher employment and self-reliant living.

### 4.6. Direction of Future Policy

It is true that there is increasing new policy action for single mothers and changing social norms in Korea [18]. However, we did not find evidence of such change and public effort in terms of the success in becoming self-reliant in the experiences of single mothers from the FGD. Because the current single-parent family policy has been operated as a subsector of poverty policy, the income-based eligibility requirements, without assessment of the unique needs of the single-parent family, might have unintended consequences. Still, the current social services/policies for single-parent families consider unwed single mothers as an unhealthy or informal family type, so that positive policy action has not happened. To increase the birth rate, the Korean government should separate single-parent family policy from poverty or healthy family policy. Regardless of family type, income level, and marriage status, much more strengthening of the quality of parenting and public childcare systems is a better direction for policy making.

In the future, the policy evaluation of national childcare systems is required with statistical evidence. The systematic assessment of single mothers’ status is necessary as a part of universal population policy rather than targeted poverty strategies. Future studies can identify blind spots in terms of the single-parent family or other vulnerable family groups. Unlike previous studies narrowly focusing on personal experiences of single mothers [19,20], this study qualitatively explored the direct experiences of single mothers regarding social policy and services in healthcare, childcare, housing, employment, and income security. This study contributes to building a foundation of future policy evaluation guidelines and key themes for the policy framework design.

## 5. Conclusions

This study confirmed that there are discrepancies between the direction of social services/policies and single mothers’ needs. Single mothers had difficulties in five life sectors: role of health care providers, childcare, housing, employment, and income security. These occurred during significant periods of single mothers’ lives including pregnancy, childbirth, and parenting. Thus, the Korean government and society should make an effort to reflect single mothers’ needs in policy planning based on a systematic life-course approach: pregnancy, childbirth, and parenting. The new policy can intervene with respect to current blind spots in services/policies for single mothers, and provide a social safety net to strength single mothers’ self-reliance and children’ social security in the long term.

## Figures and Tables

**Table 1:** Study setting and FGD guidelines.

Category	Contents
Participants	Single mothers currently raising children and living in the community
Location	The Center for Qualitative Research, Gallup Korea in Seoul, Korea
# of Groups	1 group (7 participants)
Date	27 September 2014
Method	Focus Group Discussion
**FGD Guideline**	
Pregnancy	What did you need when you recognized that you were pregnant? How was your living condition during pregnancy? What difficulties did you have during pregnancy? How and what social services did you use during pregnancy? What are (were) the most essential social services and supports for single mothers during pregnancy?
Childbirth	What did you consider when you decided on childbirth? What difficulties did you have during childbirth? What social services did you use when you gave birth, and how did you use them? What are (were) the most essential social services and supports for single mothers during childbirth?
Parenting	When and how did you choose to be a parent? How have you been doing since childbirth? How is your current parenting experience? What difficulties do you have in parenting? What kinds of social services do you use for parenting? When did you experience the most difficult part of parenting? What do you think are the most important things for parenting? What do you think about current weaknesses of social services and policies for single mothers? Based on your experience, what kinds of social services and policies should be promoted first? What are the most supportive things for your current life?

**Table 2:** Participants’ characteristics.

Age at Childbirth	Education at Pregnancy	Highest Level of Education	Current Job Status	Age of Child	Current Childcare	Housing
29	Graduated high school	Enrolled university student	In job training	5	Daycare center	Public housing
31	Graduated college	Enrolled university student	Office worker	3	Grandmother’s help	Public housing
29	Graduated college	Graduated college	Office worker	5	Daycare center	Public housing
15	Dropped out of high school	Enrolled university student	Job-seeking (Previous office worker)	6	Daycare center	Public housing
24	Enrolled university student	Graduated university	Part-time worker	4	Daycare center	Public housing
17	Graduated middle school	Enrolled university student	Office worker	3	Daycare center	Public housing
24	Graduated university	Graduated university	Service industry worker	6	Daycare center	Dormitory of office

**Table 3:** Welfare benefits for single-parent families.

Type		Single Parent over 25 Years Old	Single Parent under 25 Years Old
Pregnancy	Medical expenses	$500 (additional $1,200 if a mother is under 18 years old)	
Childbirth	Maternity expenses	$600 (household income < 43% of the standard median income)	
Parenting	A child under 15 years old	$130/month (household income <52% of the standard median income)	$180/month (a household income <60% of the standard median income)
	A child under 6 years old	Additional $50/month (household income <52% of the standard median income)	
	A child in welfare facilities	$50/month a household + additional social services and housing, etc.	

Note: The standard median income for 2-member households per month in Korea in 2018 = KRW 2,847,097 (around $2850).

## Appendix A

(1) In this paper, the term single mothers represents unwed mothers implying an involuntary, failed relationship with their children’s fathers without marriage [6,21]. In other words, they are single by chance, but mothers by choice.
(2) The eligibility for welfare benefits in the Act only includes single-parent families who have an income not exceeding 52% of the median income in Korea (KRW 1,480,490, $1500, as of 2018 for 2-member households per month) [22]. Adolescent unmarried mothers aged 24 or younger are provided with KRW 180,000 ($180) per month, when they meet the income criterion by not exceeding 60% of the median income in Korea (KRW 1,708,258, $1700, as of 2018 for 2-member households), except those who received national basic livelihood security.
(3) According to the estimation of the Korean Women’s Development Institute in 2009, the largest group of single mothers were in their 20s (52.7%) and the next largest group were teenagers (30.6%) [23].
